# Effects of lipopolysaccharide infusion on feed intake, apparent digestibility, rumen fermentation and microorganisms of young Holstein bulls fed diets with different ratios of lysine and methionine

**DOI:** 10.3389/fvets.2024.1523062

**Published:** 2025-01-06

**Authors:** Huiyao Wang, Hongyun Liu, Shijia Pan, Zhicong Ma, Yanming Wang, Jianxin Liu, Chong Wang, Zhigao An

**Affiliations:** ^1^College of Animal Science and Technology & College of Veterinary Medicine, Key Laboratory of Applied Technology on Green-Eco-Healthy Animal Husbandry of Zhejiang Province, Zhejiang A&F University, Hangzhou, Zhejiang, China; ^2^College of Animal Sciences, Zhejiang University, Hangzhou, Zhejiang, China; ^3^Kemin (China) Technologies Co. Ltd., Zhuhai, China

**Keywords:** lipopolysaccharide, lysine, methionine, young Holstein bulls, rumen microorganism

## Abstract

The aim of this experiment was to investigate the effects of intravenous infusion of lipopolysaccharide (LPS) and feeding different ratios of lysine (Lys) and methionine (Met) on feed intake, apparent digestibility, rumen fermentation and microorganisms in young Holstein bulls. Five seven-month-old Holstein bulls with similar body weights (279 ± 42 kg) were selected and subjected to a 5 × 5 Latin square experiment. The control group (CON) was fed with basal diet and the ratio of Lys to Met in the diet was adjusted to 3.0: 1. The experimental groups were received LPS infusion while being fed the basal diet (TRT1), along with LPS infusion and the addition of rumen-protected lysine (RPL) and rumen-protected methionine (RPM) to make the ratio of Lys to Met to 2.5:1 (TRT2), 3.0:1 (TRT3) and 3.5: 1 (TRT4), respectively. The LPS jugular infusion dose was set at 0.01 μg/kg body weight on days 1–3 and 0.05 μg/kg body weight on days 4–7. The trial was conducted over five periods, consisting of a 7-day trial period and a 6-day interval. The results indicated that there were no significant effects of LPS infusion on feed intake and apparent digestibility in young Holstein bulls fed different ratios of Lys and Met (*p* > 0.05). The treatment had no significant effects on the pH and total volatile fatty acids (*p* > 0.05). Compared with CON, the acetate content in the experimental groups exhibited an increasing trend (*p* = 0.066), while the content of NH_3_-N decreased significantly (*p* < 0.05). LPS infusion had no significant effect on rumen microorganisms at either the species or phylum level (*p* > 0.05). However, feeding different ratios of Lys and Met could significantly increasing the abundance of *Oribacterium* (*p* < 0.05) and tended to increase the abundance of *norank_f__norank_o__RF_39* at the genus level (*p* = 0.087). These findings suggest that adding RPL and RPM into the diet may enhance the rumen environment in young Holstein bulls. Under the conditions of this experiment, adding RPL and RPM can mitigate the negative effects associated with LPS infusion, with an optimal ratio of Lys and Met is 3.0:1.

## 1 Introduction

Lipopolysaccharide (LPS) is a common substance that induces inflammation, which can lead to weakened immunity, decreased feed intake, and substandard product quality in livestock and poultry. In the process of protein digestion and utilization of dairy cattle, the rumen characteristics lead to low protein utilization efficiency (1). Amino acids (AA) are the fundamental building blocks of proteins, and the essence of protein nutrition lies in amino acid nutrition. By reducing the protein level in the diet, an appropriate amount of AA can be provided to effectively meet the growth requirements of dairy cattle (2). The overall concentration of AA in the body is strictly regulated through transport, metabolism, and protein biosynthesis/degradation. Organs work together to achieve systemic amino acid homeostasis, and carried out through intestinal absorption and peptides and renal reabsorption. The liver metabolizes AA and muscles participate in protein biosynthesis and degradation, and the nervous and endocrine systems regulate this transport and metabolism (3). Host-rumen microbiota interaction is an important component and complex ecosystem of many physiological processes. Ruminant can consume large amounts of forage and convert it into products, mainly relying on some bacteria in the rumen to secrete cellulase, which could decompose partly cellulose as an energy source for the body (4).

Lysine (Lys) and methionine (Met) are the two primary limiting essential AA for ruminant growth, playing a crucial role in supporting gastrointestinal function and the gut-related immune system. Met promotes the growth and development of immune organs, enhances the proliferation of immune cells, and facilitates the synthesis of immune-related compounds, thereby regulating the body’s immune response (5). Lys induces antioxidant enzymes to prevent oxidative stress through the Nrf_2_ pathway, improves the repair capabilities of proteins and DNA, and enhances the cellular redox state of intestinal cells, thus bolstering the body’s antioxidant capacity (6). Junior et al. (7) found the supplementation of rumen-protected methionine (RPM) could enhance the performance of dairy cows during peak lactation by increasing amino acid metabolism. It has been reported that RPM supplementation could alleviate heat stress (8). Moreover, the addition of Lys significantly improved immune function in dairy cows (9). Although RPM has been rumen protected, adding RPM could enhance the rumen environment of cows (10). Therefore, the purpose of this study is to evaluate the effects of lipopolysaccharide (LPS) infusion on feed intake, apparent digestibility, rumen fermentation and microorganisms in young Holstein bulls fed diets with different ratios of Lys and Met. This experiment can provide theoretical support for adjusting the amino acid structure of the diet to alleviate stress in dairy cows, which is of great significance for practical production.

## 2 Materials and methods

### 2.1 Animal, diet and design

This study was conducted from October to December 2023 in a dairy farm and approved by the Experimental Animal Ethics Committee of Zhejiang A&F University (ZAFUAC202448). Five seven-month-old Holstein bulls with similar body weights (279 ± 42 kg) were selected for the study. The dietary composition and nutritional components are shown in Table 1. A 5 × 5 Latin square design was employed for the experiment. The control group (CON) was fed a basal diet with Lys to Met ratio of 3.0:1, and the experimental groups received the with basal diet and infused LPS (TRT1), infused LPS and added rumen-protected lysine (RPL) and RPM in the diet, resulting in ratios of 2.5:1 (TRT2), 3.0:1 (TRT3), and 3.5:1 (TRT4), respectively. LPS (*E. coli* O111:B4, L2630, 297–473-0) was sourced from Sigma-Aldrich. RPL and RPM were obtained from Kemin Industries, Inc. The rumen absorption rates of RPL and RPM are 65% and 50%, respectively, while the small intestine absorption rate is greater than 80%. To avoid excessive drug stimulation, the infusion was adjusted from a low dose to a high dose. The concentration of LPS solution infused into the jugular vein was 0.01 μg/kg BW on days 1–3 and 0.05 μg/kg BW on days 4–7. The trial was conducted over five periods, each lasting 13 days, which included a 7-day experimental period followed by a 6-day rest period. During both the rest and experimental periods, the bulls were fed twice daily and had unrestricted access to water.

**Table 1:** Dietary and nutrition composition levels.

Dietary components%		Nutrient level%	
Wheat straw	13.04	DM	92.91
Peanut seedlings	26.08	CP	14.09
Corn silage	34.78	Ash	10.17
Corn	4.57	EE	4.75
Soybean meal	10.43	NDF	34.14
Brewer’s grains	6.52	ADF	29.73
Bran	3.26		
^1^Premix	0.44		
Stone powder	0.44		
NaCl	0.22		
Dicalcium phosphate	0.22		
Total	100		

1 Premix/kg: 580 kIU vitamin A; 220 kIU vitamin d3; 5.76 kIU vitamin e; 3,200 mg zinc; 2,560 mg manganese; 2,400 mg iron; 1,600 mg copper; 55 mg iodine; 20 mg cobalt; 50 mg selenium.

On the day before the start of each period, 1 L of infusion solution was prepared according to their weight and stored at 4°C. The solution was allowed to return to room temperature before infusion. A constant flow pump was used for the infusion, which lasted approximately 6 h. The bulls were arranged in order, and a central venous catheter was inserted into the jugular vein. The catheters were flushed with saline before infusion and promptly sealed with heparin sodium to prevent blockage due to blood coagulation. The surgical site for each trial was not on the same side as the previous one, allowing sufficient time for wound recovery.

### 2.2 Sample collection and determination

#### 2.2.1 Sample collection

Each bull was independently fed twice a day, the remaining feed was weighed, the daily feed intake of each bull was calculated, and the feed intake of each bull was calculated according to the treatment. On the last 3 days of each period, spot collection of feces every 6 h. The first collection on the second day would be collected 2 h in advance and 4 h in advance on the third day. Fecal samples would be pooled by periods and bulls. The feces were then mixed according to the cattle at the end of each period and stored at −80°C. After a 7-day experimental phase, 50 mL of rumen fluid was collected before morning feeding, discarding the first 200 mL to avoid saliva contamination. The rumen fluid was filtered through four layers of sterile gauze, and the pH was immediately measured. The sample was divided into 2 mL centrifuge tubes and stored at −80°C for microbiological testing. The rumen microbiota was analyzed by Shanghai Meiji Biomedical Technology Co., Ltd.

#### 2.2.2 DNA extraction, PCR amplification and Illumina MiSeq sequencing

Total microbial genomic DNA was extracted using the E.Z.N.A.® soil DNA Kit (Omega Bio-tek, Norcross, GA, U.S.) according to manufacturer’s instructions. The quality and concentration of DNA were determined by 1.0% agarose gel electrophoresis and a NanoDrop2000 spectrophotometer (Thermo Scientific Inc., USA). The hypervariable region V3-V4 of the bacterial 16S rRNA gene were amplified with primer pairs338F (5’-ACTCCTACGGGAGGCAGCAG-3′) and 806R (5’-GGACTACHVGGGTWTCTAAT-3′), using the following conditions: 3 min of denaturation at 95°C, 27 cycles of 30 s at 95°C, 30 s of annealing at 55°C, and 45 s of elongation at 72°C, and a final extension for 10 min at 72°C (11, 12). Each PCR experiment used a 20-μL reaction mixture containing 4 μL of 5 × FastPfu buffer, 2 μL of 2.5 mM deoxynucleoside triphosphates, 0.8 μL of each primer (5 M), 0.4 μL of FastPfu polymerase, and 10 ng of template DNA. The PCR product was extracted from 2% agarose gel and purified using the PCR Clean-Up Kit (YuHua, Shanghai, China) according to manufacturer’s instructions and quantified using Qubit 4.0 (Thermo Fisher Scientific, USA). Purified amplicons were pooled in equimolar amounts and paired-end sequenced on an Illumina Nextseq2000 platform (Illumina, San Diego, USA) according to the standard protocols by Majorbio Bio-Pharm Technology Co. Ltd. (Shanghai, China).

#### 2.2.3 Sample determination

Rumen fluid was centrifuged at 500 × g at 4°C for 15 min. Subsequently, 3 mL of the supernatant was mixed with 1 mL of 77.6% trichloroacetic acid solution, vortex-mixed, and divided into two portions. One placed in an ice bath for 45 min and the other centrifuged at 27,000 × g for 15 min. Microbial protein (MCP) was measured using the Coomassie brilliant blue method with a multifunctional enzyme-labeled instrument (Tecan Infinite 200Pro, Tecan Group, Switzerland) at a wavelength of 595 nm. The collected rumen fluid was centrifuged at 1,000 × g at 4°C for 10 min. 1 mL of the supernatant was transferred into a centrifuge tube, and 0.1 mL of 25% metaphosphate was added. The mixture was then placed in an ice bath for 30 min, followed by centrifugation at 1,000 × g at 4°C for 15 min. The supernatant was analyzed for acetate, propionate, butyrate, isobutyrate, valerate, and isovalerate using a gas chromatograph (6,890 N, Agilent, USA). The concentration of NH_3_-N in rumen fluid was determined using indophenol colorimetry (13).

The contents of dry matter (DM) and crude protein (CP) in both feed and feces were determined using the AOAC method (1990) (14). Additionally, the levels of acid detergent fiber (ADF) and neutral detergent fiber (NDF) were assessed following the method established by Van Soest et al. (15).

### 2.3 Statistical analysis

The feed intake, apparent digestibility, rumen fermentation and microbial composition were all sorted by Excel 2022. Feed intake, apparent digestibility, rumen fermentation indexes and microbial composition were analyzed by PROC GLM program in SAS9.4 statistical software. Treatment and period were fixed effects in the model, while bulls were random effects. Test results are presented as mean and mean standard error (SEM). *p* < 0.05 indicated significant difference, and 0.05 ≤ *p* < 0.10 indicated trend of difference.

## 3 Results

### 3.1 Feed intake

The effects of LPS infusion on feed intake of young Holstein bulls fed diets with different ratios of Lys and Met is shown in Table 2. During the whole period, treatment had no significant effect on feed intake of young Holstein bulls (*p* > 0.05).

**Table 2:** Effect of lipopolysaccharide infusion on feed intake of young Holstein bulls fed diets with different ratios of Lys and Met.

Items	Treatments					SEM	*p*-value
	CON	TRT1	TRT2	TRT3	TRT4		
Mean daily feed intake kg/d DM	14.91	13.97	15.19	14.96	14.95	0.709	0.423

CON = basal diet, the ratio of Lys to Met is 3.0:1; TRT1 = LPS infusion and basal diet, the ratio of Lys to Met is 3.0:1; TRT2 = LPS infusion, basal diet added rumen-protected amino acids, the ratio of Lys to Met is 2.5:1; TRT3 = LPS infusion, basal diet added rumen-protected amino acids, the ratio of Lys to Met is 3.0:1; TRT4 = LPS infusion, basal diet added rumen-protected amino acids, the ratio of Lys to Met is 3.5:1; SEM = standard error of the mean.

### 3.2 Apparent digestibility

Effect of LPS infusion on apparent digestibility of nutrients in young Holstein bulls fed diets with different ratios of Lys and Met are shown in Table 3. Treatment had no significant effect on the apparent digestibility of DM, CP, NDF and ADF among groups (*p* > 0.05).

**Table 3:** Effects of lipopolysaccharide infusion on apparent digestibility of young Holstein bulls fed diets with different ratios of Lys and Met.

Items	Treatments					SEM	*p*-value
	CON	TRT1	TRT2	TRT3	TRT4		
DM	64.02	61.44	62.70	66.08	64.99	2.939	0.946
CP	71.72	68.42	70.51	73.28	71.90	1.655	0.840
NDF	50.86	47.31	58.51	58.90	54.38	7.680	0.855
ADF	42.89	38.25	54.01	56.00	53.58	5.637	0.107

CON = basal diet, the ratio of Lys to Met is 3.0:1; TRT1 = LPS infusion and basal diet, the ratio of Lys to Met is 3.0:1; TRT2 = LPS infusion, basal diet added rumen-protected amino acids, the ratio of Lys to Met is 2.5:1; TRT3 = LPS infusion, basal diet added rumen-protected amino acids, the ratio of Lys to Met is 3.0:1; TRT4 = LPS infusion, basal diet added rumen-protected amino acids, the ratio of Lys to Met is 3.5:1; SEM, standard error of the mean.

### 3.3 Rumen fermentation

Effects of LPS infusion on rumen fermentation in young Holstein bulls fed diets with different ratios of Lys and Met are shown in Table 4. The NH_3_-N content of CON and TRT1 was significantly higher than that of TRT3 and TRT4 (*p* < 0.05), and it is the lowest in TRT3. During the whole experimental period, the acetate content in the experimental group showed an increasing trend (*p* = 0.066) with the highest content in the TRT3, and the treatment had no significant effect on the pH, MCP, total volatile fatty acids (TVFAs), propionate, isobutyrate, butyrate, isovalerate, valerate and the ratio of acetate and propionate of rumen fluid (*p* > 0.05).

**Table 4:** Effects of lipopolysaccharide infusion on rumen fermentation of young Holstein bulls fed diets with different ratios of Lys and Met.

Items	Treatments					SEM	*p*-value
	CON	TRT1	TRT2	TRT3	TRT4		
pH	6.39	6.47	6.51	6.49	6.52	0.204	0.992
NH_3_-N, mg/L	2.36^a^	2.38^a^	1.47^ab^	0.92^b^	1.27^b^	0.349	0.029
MCP, mg/mL	1.26	1.32	1.24	1.42	1.51	0.167	0.791
TVFAs, mmol/L	85.63	96.10	87.20	103.50	100.88	5.926	0.201
Acetate, mmol/L	52.13	59.30	52.10	64.63	63.28	4.613	0.066
Propionate, mmol/L	13.81	15.55	13.85	17.24	16.67	1.392	0.340
Isobutyrate, mmol/L	5.00	5.68	5.13	6.12	5.93	0.560	0.573
Butyrate, mmol/L	6.61	7.10	6.83	7.04	6.81	0.403	0.910
Isovalerate, mmol/L	3.14	3.40	3.28	3.35	3.14	0.287	0.945
Valerate, mmol/L	4.94	5.07	5.00	5.11	5.06	0.154	0.943
Acetate: Propionate	3.85	3.83	3.92	3.75	3.80	0.236	0.989

CON = basic diet, the ratio of Lys to Met is 3.0:1; TRT1 = LPS infusion and basic diet, the ratio of Lys to Met is 3.0:1; TRT2 = LPS infusion, basic diet added rumen-protected amino acids, the ratio of Lys to Met is 2.5:1; TRT3 = LPS infusion, basic diet added rumen-protected amino acids, the ratio of Lys to Met is 3.0:1; TRT4 = LPS infusion, basic diet added rumen-protected amino acids, the ratio of Lys to Met is 3.5:1; SEM, standard error of the mean. ab means in a row that do not have a common superscript letter differ significantly (*p* < 0.05); ab means in a row that have a common superscript letter no differ significantly (*p* > 0.05).

### 3.4 Rumen microorganisms

**Figure 1:**
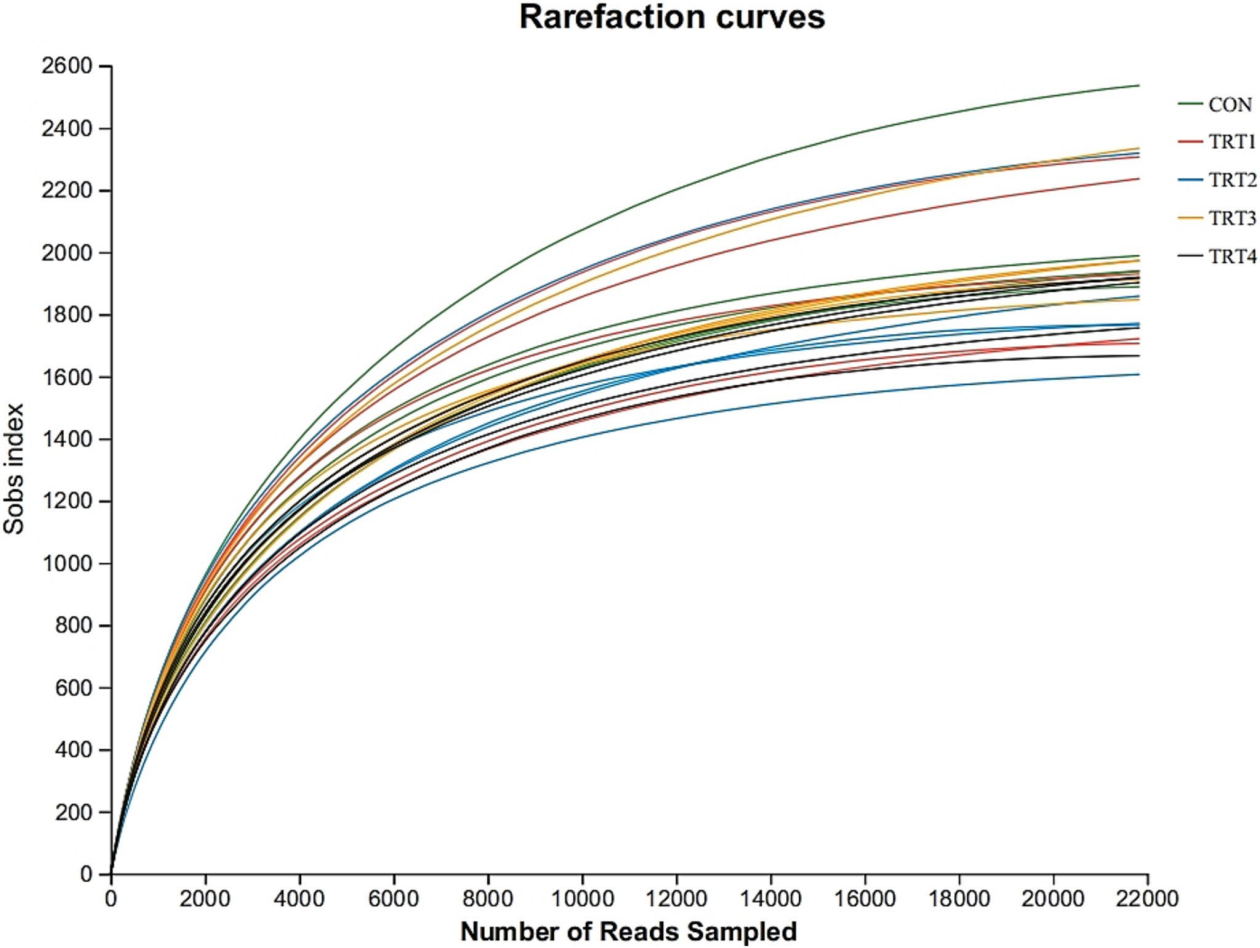
Sample dilution curve in different groups. CON = basal diet, the ratio of Lys to Met is 3.0:1; TRT1 = LPS infusion and basal diet, the ratio of Lys to Met is 3.0:1; TRT2 = LPS infusion, basal diet added rumen-protected amino acids, the ratio of Lys to Met is 2.5:1; TRT3 = LPS infusion, basal diet added rumen-protected amino acids, the ratio of Lys to Met is 3.0:1; TRT4 = LPS infusion, basal diet added rumen-protected amino acids, the ratio of Lys to Met is 3.5:1.

As shown in Figure 1, rumen microbial dilution correlation curve tended to be stable when the measured effective sequence number reached 22,000, indicating that the data amount was reasonable.

**Figure 2:**
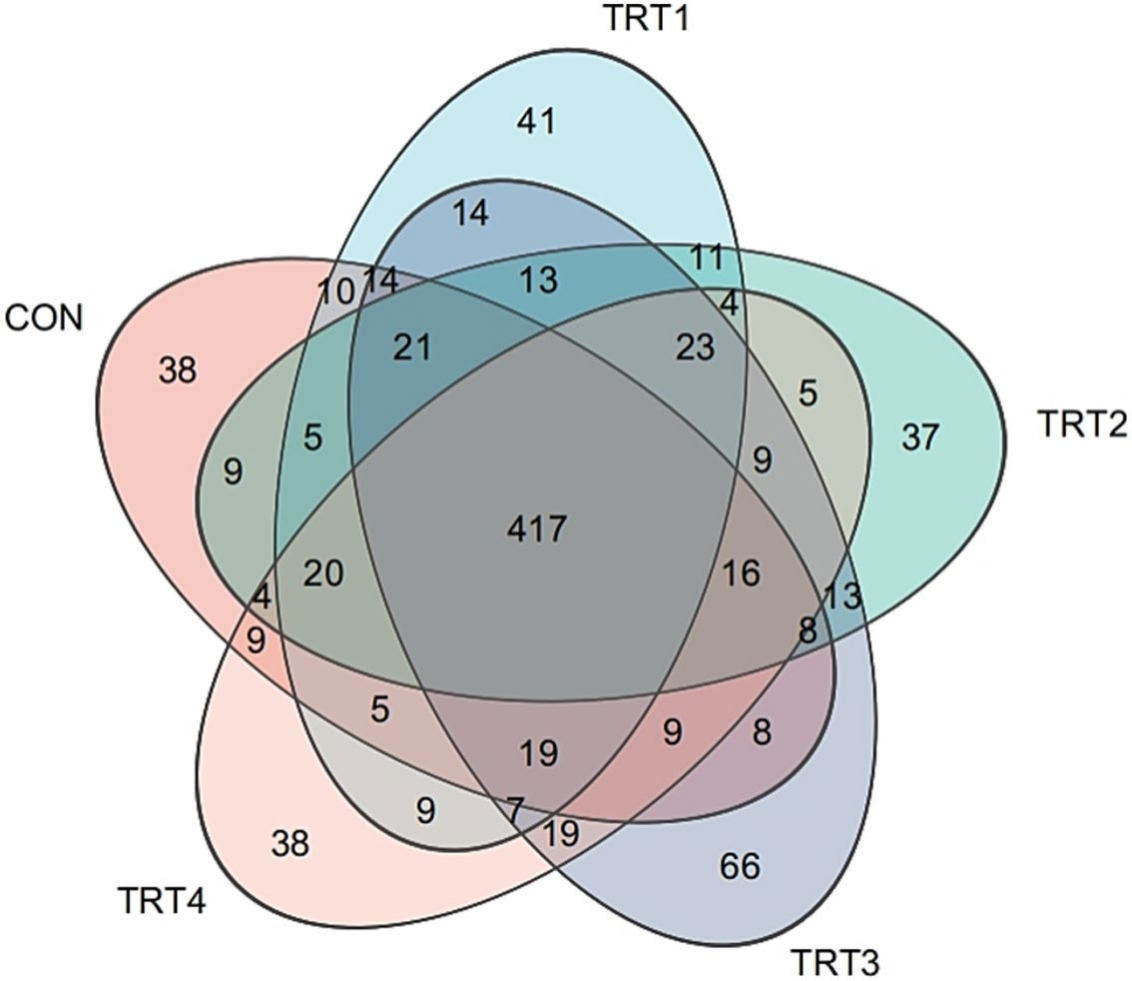
ASV level venn diagramin different groups. CON = basal diet, the ratio of Lys to Met is 3.0:1; TRT1 = LPS infusion and basal diet, the ratio of Lys to Met is 3.0:1; TRT2 = LPS infusion, basal diet added rumen-protected amino acids, the ratio of Lys to Met is 2.5:1; TRT3 = LPS infusion, basal diet added rumen-protected amino acids, the ratio of Lys to Met is 3.0:1; TRT4 = LPS infusion, basal diet added rumen-protected amino acids, the ratio of Lys to Met is 3.5:1.

A total of 25 rumen samples were collected for DNA extraction and 16S rRNA sequencing. As shown in Figure 2, Venn diagram showed the number of common and specific ASVs in the rumen of bulls in different groups: the five groups had 417 ASVs in common, including 38 specific ASVs in the CON, 41 specific ASVs in the TRT1, 37 specific ASVs in the TRT2, 66 specific ASVs in the TRT3 and 38 specific ASVs in the TRT4.

Alpha diversity mainly reflects the diversity of species. As shown in Table 5, goods-coverage indexes were all above 0.9, which indicated that species diversity and community structure could be well reflected. There was no significant effect of LPS and feeding with different ratios of Lys and Met on the alpha diversity of rumen microorganisms in bulls (*p* > 0.05).

**Table 5:** Effects of lipopolysaccharide infusion on alpha diversity of young Holstein bulls fed diets with different ratios of Lys and Met.

Items	Treatments					SEM	*p*-value
	CON	TRT1	TRT2	TRT3	TRT4		
Chao1	2,140	2067	1926	2,115	1923	89.768	0.203
Simpson’s index	2058	1980	1863	2007	1832	77.045	0.109
Shannon index	6.79	6.72	6.51	6.73	6.60	0.105	0.153
Goods-coverage	0.99	0.99	0.99	0.99	0.99	0.001	0.570

CON = basal diet, the ratio of Lys to Met is 3.0:1; TRT1 = LPS infusion and basal diet, the ratio of Lys to Met is 3.0:1; TRT2 = LPS infusion, basal diet added rumen-protected amino acids, the ratio of Lys to Met is 2.5:1; TRT3 = LPS infusion, basal diet added rumen-protected amino acids, the ratio of Lys to Met is 3.0:1; TRT4 = LPS infusion, basal diet added rumen-protected amino acids, the ratio of Lys to Met is 3.5:1; SEM, standard error of the mean.

As shown in Table 6, there was no significant effect of LPS infusion on the species difference of rumen microorganisms at the phylum level in bulls which fed diets with different ratios of Lys and Met (*p* > 0.05). Table 7 showed that feeding diets with different proportions of Lys and Met could significantly increase the content of *Oribacterium* in the rumen (*p* < 0.05), with that highest *Oribacterium* content in bulls treated with TRT3. Additionly, *norank_f__norank_o__RF39* showed an increasing trend (*p* = 0.078) at genus level.

**Table 6:** Comparison of species differences in rumen microorganisms at phylum level in young Holstein bulls infused lipopolysaccharide and fed diets with different Lys and Met ratios (relative abundance >0.5%).

Items	Groups					SEM	*p*-value
Species name	CON	TRT1	TRT2	TRT3	TRT4		
*Bacteroidota*	50.29	48.79	48.25	41.45	55.66	5.267	0.359
*Firmicutes*	42.43	41.19	43.57	50.58	38.14	4.910	0.423
*Patescibacteria*	3.60	5.89	3.73	3.51	2.46	1.178	0.255
*Spirochaetota*	1.28	1.39	1.49	0.80	1.32	0.219	0.426
*Actinobacteriota*	0.56	0.70	0.73	1.11	1.12	0.213	0.148

CON = basal diet, the ratio of Lys to Met is 3.0:1; TRT1 = LPS infusion and basal diet, the ratio of Lys to Met is 3.0:1; TRT2 = LPS infusion, basal diet added rumen-protected amino acids, the ratio of Lys to Met is 2.5:1; TRT3 = LPS infusion, basal diet added rumen-protected amino acids, the ratio of Lys to Met is 3.0:1; TRT4 = LPS infusion, basal diet added rumen-protected amino acids, the ratio of Lys to Met is 3.5:1; SEM, standard error of the mean.

**Table 7:** Comparison of species differences in rumen microorganisms at genus level in young Holstein bulls fed diets with different ratios of Lys and Met by lipopolysaccharide infusion.

Items	Treatments					SEM	*p-*value
	CON	TRT1	TRT2	TRT3	TRT4		
*Prevotella*	26.12	21.87	23.95	15.20	30.18	4.723	0.293
*Rikenellaceae_RC9_gut_group*	7.39	9.70	7.35	8.85	8.23	1.034	0.465
*norank_f__F082*	6.20	7.41	6.29	8.39	6.21	1.060	0.526
*Christensenellaceae_R-7_group*	5.04	6.36	5.34	6.39	5.29	0.992	0.661
*NK4A214_group*	4.67	5.42	4.62	5.40	4.81	0.651	0.693
*Candidatus_Saccharimonasw*	3.18	5.73	3.41	3.21	2.30	1.150	0.225
*unclassified_c__Clostridia*	2.65	2.11	2.78	3.72	2.66	0.482	0.252
*Prevotellaceae_UCG-003*	2.76	1.92	3.45	1.26	2.94	0.777	0.341
*norank_f__norank_o__Clostridia_UCG-014*	3.26	1.91	2.50	2.49	1.71	0.477	0.156
*unclassified_f__Lachnospiraceae*	2.03	2.25	2.28	3.39	1.66	0.500	0.209
*norank_f__Bacteroidales_RF16_group*	1.62	1.69	1.06	3.18	2.07	0.864	0.374
*norank_f__norank_o__RF39*	2.10	1.47	2.01	2.31	1.26	0.283	0.078
*Lachnospiraceae_NK3A20_group*	1.52	1.35	1.41	2.12	1.75	0.528	0.723
*Ruminococcus*	1.95	1.03	1.28	1.82	1.27	0.422	0.483
*norank_f__UCG-010*	1.41	1.41	1.36	1.43	1.30	0.219	0.989
*Butyrivibrio*	0.91	0.93	1.68	1.54	1.15	0.441	0.655
*Prevotellaceae_UCG-001*	1.20	1.11	1.29	0.81	1.49	0.317	0.609
*Saccharofermentans*	0.96	1.09	1.31	1.20	0.99	0.182	0.613
*norank_f_Eubacterium_coprostanoligenes_group*	1.06	0.87	1.50	1.12	0.89	0.257	0.425
*UCG-005*	1.12	1.07	0.91	1.07	1.13	0.255	0.977
*Anaeroplasma*	1.18	1.02	1.05	1.10	0.92	0.270	0.955
*Succiniclasticum*	1.14	1.39	1.24	0.57	0.89	0.394	0.629
*Treponema*	1.05	1.23	1.20	0.62	1.12	0.189	0.202
*norank_f__Muribaculaceae*	1.02	1.02	1.02	0.73	0.82	0.156	0.564
*Oribacterium*	0.50^b^	1.10^a^	0.89^ab^	1.28^a^	0.42^b^	0.191	0.010
*unclassified_f__Prevotellaceae*	0.78	0.76	0.96	0.45	0.93	0.245	0.449
*Acetitomaculum*	0.53	0.61	0.58	0.88	0.58	0.181	0.551

CON = basal diet, the ratio of Lys to Met is 3.0:1; TRT1 = LPS infusion and basal diet, the ratio of Lys to Met is 3.0:1; TRT2 = LPS infusion, basal diet added rumen-protected amino acids, the ratio of Lys to Met is 2.5:1; TRT3 = LPS infusion, basal diet added rumen-protected amino acids, the ratio of Lys to Met is 3.0:1; TRT4 = LPS infusion, basal diet added rumen-protected amino acids, the ratio of Lys to Met is 3.5:1; SEM = standard error of the mean. ab means in a row that do not have a common superscript letter differ significantly (*p* < 0.05); ab means in a row that have a common superscript letter no differ significantly (*p* > 0.05).

## 4 Discussion

The aim of this experiment is to explore effects of lipopolysaccharide infusion on feed intake, apparent digestibility, rumen fermentation and microorganisms of young Holstein bulls fed diets with different ratios of Lys to Met. The experimental results can provide theoretical support for exploring the amino acid structure of dairy cows’ diets under chronic stress.

Feed intake is the primary factor of determining efficiency in production, and whether it is normal or not significantly impacts animal production (16). Stress is one of the main contributors to decreased feed intake in animals. Low feed intake adversely affects the rumen condition of ruminants (17–19). It has been discovered that a state of stress could lead to a significant reduction in feed intake among mice (20). It has been demonstrated that a variety of stresses including oxidative stress can lead to low feed intake in ruminants (21). Meanwhile, Kang et al. (22) reported that intraperitoneal injection of a high dose of LPS inhibited feed intake. Similar results have been found in studies of dairy cows (23). However, no difference in feed intake obtained by LPS infusion may causing by the dose in this study, which lead to varying doses of RPL and RPM did not significantly effect of young Holstein bulls. The LPS dose selected in this experiment was relatively low, as well as in order to avoid acute stress on the bulls. And a concentration gradient was used to gradually increase the infusion dose, giving the bulls enough time to adapt. This may be the reason why the feed intake decreased to a certain extent while no difference among groups.

Apparent digestibility reflects the efficiency with animals utilize feed. It has been found that incorporating RPL and RPM into the diet of yaks increased nutrient digestibility (24). Dietary addition of RPL and RPM have also been shown to promote the digestibility of CP and NDF in other ruminants (25, 26), likely because methionine or its analogs increased the abundance of cellulose-decomposing bacteria in the rumen (27), there are differences from our experimental results, which also may be related to the rumen passage rate of AA and the level of feeding management. However, the same trend has not been shown in studies of dairy cows (28, 29), which aligns with the findings of this study. Zhao et al. (30) and Lee et al. (31) found that RPM and other essential amino AA did not influence the apparent digestibility of nutrients in dairy cows’ diets, consistent with our research findings. Similarly, Odedra et al. (32) reported that supplementing RPL into the basal diet of goats did not significantly affect the apparent digestibility of DM, ADF, and NDF, consistent with the results of this study. The discrepancies in these research findings may be attributed to the use of different types of rumen-protected AA across studies, which exhibit distinct rumen protection rates, rumen microbial metabolism rates, and apparent digestibility rates.

In the normal physiological range, a lower rumen pH value is beneficial for the rumen development of growing cattle (33). However, when the pH exceeds this range, whether it becomes too acidic or too alkaline, it can lead to a decline in animal production performance, directly negatively impacting rumen fermentation. In severe cases, subacute acidosis may occur, potentially resulting in the death of cattle (34, 35). In this study, the treatments had no significant effect on the rumen pH of young Holstein bulls. Ruminants cannot directly utilize ammonia nitrogen, however, rumen bacteria possess the ability to capture free ammonia nitrogen in the rumen for MCP synthesis (36). The rumen and intestinal microflora serve as intermediaries in nutrient absorption. Through a symbiotic relationship with rumen microorganisms, all forms of dietary nitrogen sources are ultimately degraded into peptides, AA, and ammonia in the rumen. Increasing MCP yield is an effective strategy for enhancing ruminant performance and reducing protein feed waste (37). Macelline et al. (38) reported that broilers fed a low-protein diet supplemented with synthetic AA could sustain growth performance and intestinal integrity while reducing nitrogen excretion. In ruminant, low-protein diet supplemented with RPM promoted rumen digestibility and the production of volatile fatty acids (39). Additionally, Van et al. (40) discovered that supplementing RPM and RPL in a low-protein diet can improve production performance, maintain nitrogen balance, and enhance nitrogen utilization efficiency in dairy cows. However, some studies also showed that the addition of Lys and Met had no effect on rumen fermentation parameters and only improved nitrogen use efficiency (29, 41). Under these experimental conditions, feeding different proportions of RPL and RPM decreased the concentration of NH_3_-N in the rumen, corroborating the aforementioned findings. Löest et al. (42) found that cattle exposed to LPS exhibited reduced recyclable nitrogen, leading to increased AA oxidation. The observed rise in ruminal NH_3_-N concentration 24 h after LPS infusion may be attributed to an imbalance between tissue AA supply and immune system AA demand, or due to increased AA oxidation to support other metabolic functions (43). It indicated supplementation of ruminal AA helps maintain this balance and improves nitrogen utilization (44, 45). This suggests that under LPS infusion conditions, incorporating RPL and RPM into the diet of young Holstein bulls can enhance nitrogen utilization efficiency, reduce nitrogen excretion, improve NH_3_-N utilization efficiency, and optimize their rumen fermentation status, and the optimal ratio of Lys and Met in diet is 3.0: 1.

LPS can cause rumen microbial dysbiosis in cows (46). One of the purposes of this study is to evaluate the effects of LPS infusion on rumen microorganisms in young Holstein bulls fed diets with different ratios of Lys and Met. In this study, feeding diets with different proportions of Lys and Met had no significant effect on rumen microbial diversity at the phylum level and the alpha diversity of rumen microbiota, which aligns with the results reported by the previous studies (47, 48). At the genus level, different proportions of Lys and Met were found to increase the abundance of *norank_f__norank_o__RF39* and *Oribacterium*, maintained rumen homeostasis. The abundance of *norank_f__norank_o__RF39* showed a significant positive correlation with NDF digestibility (49). Therefore, the higher NDF in TRT3 compared to other groups may be related to the increase in *norank_f__norank_o__RF39*. *Oribacterium* is a strict anaerobic bacterium that plays a crucial role in synthesizing acetate, propionate, and butyrate for its host (50, 51). Additionally, enhanced fiber degradation by the action of *Oribacterium* was reported in the previous study (52). These TVFAs can be secreted and absorbed by the intestinal tract as nutrients and are also essential for maintaining the host’s health. These changes may lead to the degradation of nutrients and improve utilization efficiency in the rumen. The increase in rumen acetic acid concentration in TRT3 may be related to the increase in *Oribacteria* abundance.

## 5 Conclusion

In conclusion, adding RPL and RPM in diets for young Holstein bulls which infused by LPS has no effect on feed intake and apparent digestibility, but can reduce the concentration of NH_3_-N in the rumen, the lowest one is when Lys to Met is 3.0:1. Meanwhile, it can increase the abundance of *norank_f__norank_o__RF39* and *Oribacterium* at genus level, the highest one is when Lys to Met is 3.0:1, helping to maintain a stable state of rumen microflora. Thus, it has the best improvement effect on the rumen of young Holstein bulls infused by LPS whenthe ratio of Lys to Met in the diet was 3.0:1.

## Data Availability

The raw data supporting the conclusions of this article will be made available by the authors, without undue reservation.
